# Outbreak-Related Porcine Epidemic Diarrhea Virus Strains Similar to US Strains, South Korea, 2013

**DOI:** 10.3201/eid2007.140294

**Published:** 2014-07

**Authors:** Sunhee Lee, Changhee Lee

**Affiliations:** Kyungpook National University, Daegu, South Korea

**Keywords:** porcine epidemic diarrhea, PED, porcine epidemic diarrhea virus, PEDV, pigs, South Korea, US strain–like strains, outbreaks, viruses, order Nidovirales, family Coronaviridae, genus Alphacoronavirus genus, economic loss, pork industry

## Abstract

In late 2013, outbreaks of porcine epidemic diarrhea virus (PEDV) infection recurred in South Korea. Genetic and phylogenetic analyses showed that isolates from the outbreaks were most closely related to emergent US strains of PEDV. These US strain–like PEDV variants are prevalent in South Korea and responsible for recent outbreaks in the country.

Porcine epidemic diarrhea (PED), a devastating swine disease, is characterized by watery diarrhea, followed by dehydration, and a high death rate among suckling pigs (*1*,*2*). The disease was first recognized in England in 1971 (*3*), and since then, outbreaks have been reported in Europe, Asia, and recently, the United States (*4*–*7*). The causative agent of this disease, PED virus (PEDV), is a member of the order *Nidovirales*, family *Coronaviridae*, genus *Alphacoronavirus* (*2*,*8*). The virus first emerged in South Korea in 1992 (*9*), and PED outbreaks subsequently occurred every year until early 2010, causing economic losses to the pork industry. However, after South Korea experienced severe outbreaks of foot-and-mouth disease in 2010–2011, the prevalence of PEDV infections was low and only sporadic outbreaks occurred. This decline in PED epidemics likely resulted from the culling of >3 million pigs in South Korea during the 2010–2011 foot-and-mouth disease outbreaks. However, starting in late 2013, outbreaks of PED increased remarkably and swept rapidly across the country. To determine the origin and diversity of the PEDVs responsible for the ongoing outbreaks in South Korea, we sought to determine the full-length sequences of the spike proteins of field isolates and the complete genome sequence of a representative strain.

## The Study

**Figure 1:**
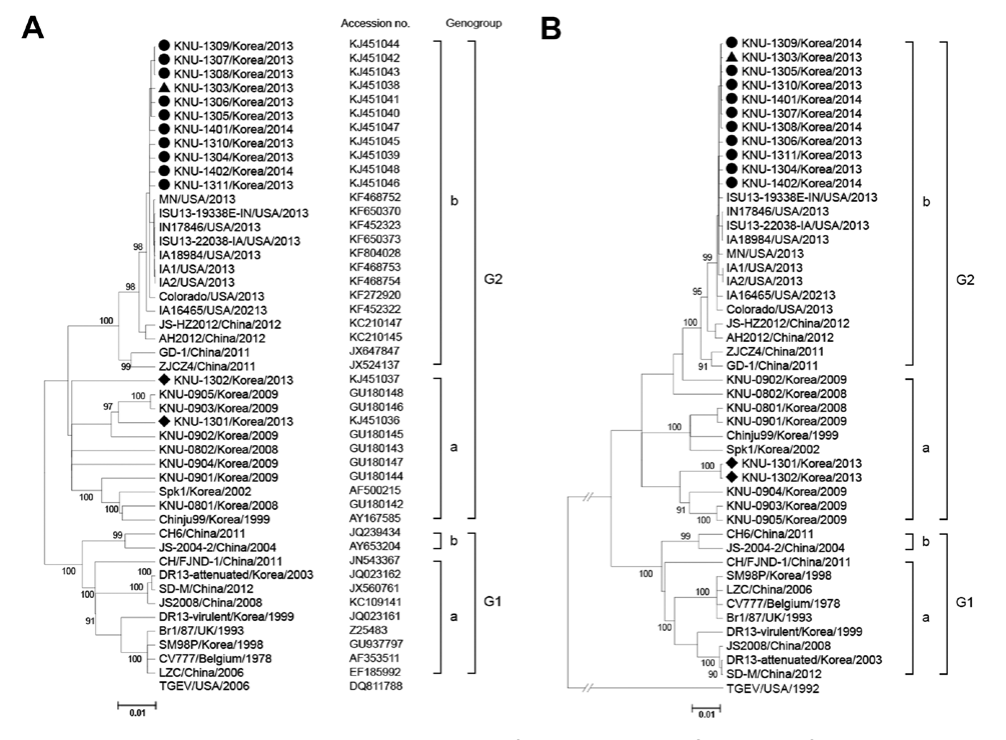
Phylogenetic analyses based on the nucleotide sequences of the spike gene (A) and S1 portion (B) of porcine epidemic diarrhea virus (PEDV) strains. A putative similar region of the spike protein of transmissible gastroenteritis virus (TGEV) was included as an outgroup in this study. Multiple-sequencing alignments were performed by using ClustalX (http://www.clustal.org/), and the phylogenetic tree was constructed from the aligned nucleotide sequences by using the neighbor-joining method. Numbers at each branch represent bootstrap values >50% of 1,000 replicates. Names of the strains, countries and years of isolation, GenBank accession numbers, and genogroups and subgroups proposed in this study are shown. Solid circles indicate the strains from South Korea from this study that are similar to US PEDV strains; solid triangle indicates the early 2013 strain that is similar to the US PEDV strains; solid diamonds indicate the early 2013 strains that are similar to the previous PEDV strains from South Korea. Scale bars indicate nucleotide substitutions per site.

During December 2013–January 2014, specimens of small intestine or feces were collected from 10 pigs that had watery diarrhea; each of the pigs lived at a different swine farm in South Korea. All samples were prepared as 10% suspensions as described elsewhere (*10*) and subjected to reverse transcription PCR using a Transmissible Gastroenteritis and Porcine Epidemic Diarrhea Detection Kit (iNtRON Biotechnology, Seongnam, South Korea) according to the manufacturer’s protocol. The full-length spike glycoprotein sequences of 10 PEDVs identified from the pigs were subsequently determined as previously described (*10*) and deposited in GenBank under the accession numbers shown in Figure 1. In addition, the complete genome of a PEDV strain, KNU-1305, was sequenced and analyzed. The 5′ and 3′ ends of the KNU-1305 genome were determined by rapid amplification of cDNA ends as described elsewhere (*11*). Ten overlapping cDNA fragments were generated to encompass the entire genome, pooled in equimolar amounts, and subjected to next-generation sequencing as previously described (*12*); the sequencing reads were assembled by using complete PEDV reference genomes from GenBank (*13*,*14*). The KOR/KNU-1305/2013 PEDV sequence data were deposited in GenBank under accession no. KJ662670. The sequences of 46 fully sequenced spike genes and the 21 complete genomes of PEDV strains were independently used in sequence alignments and phylogenetic analyses as described elsewhere (*10*).

We determined that the full-length spike genes of the PEDV strains were 9 nt longer than that of the prototype PEDV strain, CV777; this difference was caused by the presence of genetic signatures for recent PEDV field isolates as described elsewhere (*10*). The similarity between the spike genes was determined; sequence homology results are described in the Technical Appendix. Nucleotide sequence analysis showed high homology (98.8%–99.9%) among the 10 tested isolates. In contrast, the isolates all shared only 94.3%–94.7% nt sequence identity with a previously sequenced field isolate from South Korea, KNU-0801. However, the sequences of the 10 isolates were compared with those of other published PEDV strains and found to consistently share 99.2%–99.9% nt identity with recently emergent US strains.

The complete genomic sequence of KNU-1305 was determined to be 28,038 nt in length, excluding the 3′ ploy(A) tail. The complete PEDV genome of KNU-1305 shared 96.3%–99.9% nt identity with other complete PEDV genomes available in GenBank; the highest nucleotide identity (99.9%) was with US strains CO/13, IA1, IN17846, and MN. Compared with the complete genome of US strain IA1, KNU-1305 showed 49 different nucleotides: 1 each was in the 5′ untranslated region and the membrane gene, 7 were in the spike gene, and 35 and 5 were in open reading frames 1 and 3, respectively. Together, our results indicate that the PEDV isolates from South Korea were highly homologous with strains responsible for recent outbreaks in the United States.

**Figure 2:**
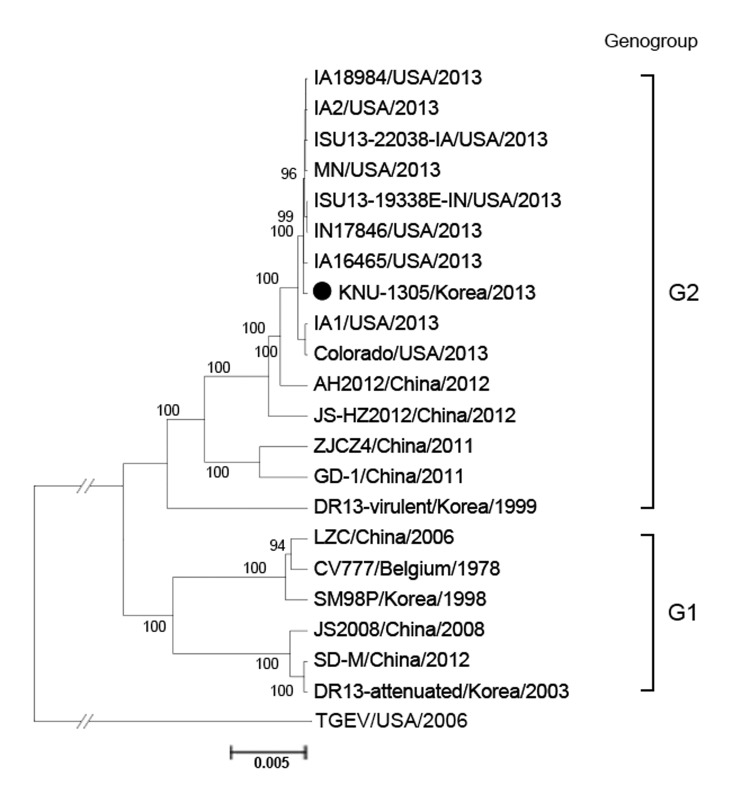
Phylogenetic analysis based on the nucleotide sequences of the full-length genomes of porcine epidemic diarrhea virus (PEDV) strains (GenBank numbers are shown in Figure 1, panel A). The complete genome sequence of transmissible gastroenteritis virus (TGEV) was included as an outgroup in this study. Numbers at each branch represent bootstrap values >50% of 1,000 replicates. Names of the strains, countries and years of isolation, and genogroups and subgroups proposed in this study are shown. Solid circle indicates the strain from South Korea, KNU-1305, that is similar to the US PEDV strains. The scale bar indicates nucleotide substitutions per site.

The full-length spike gene–based phylogenetic analysis revealed that the PEDV strains were clearly defined into 2 separate clusters, designated genogroup 1 (G1) and genogroup 2 (G2); each of the groups can be further divided into subgroups 1a, 1b, 2a, and 2b (Figure 1, panel A). All 10 PEDV strains from South Korea were classified into subgroup 2b and most closely clustered together with the recent US strains in an adjacent clade with the same subgroup, suggesting that the US strains may be the origin of the recurrence of PEDV infections in South Korea. Subsequent phylogenetic analysis of the S1 protein showed the grouping structure was the same as that in the spike gene–based tree (Figure 1, panel B). In addition, phylogenetic analysis based on the entire genome sequences demonstrated that strain KNU-1305 is grouped within the same cluster with the US stains (Figure 2).

## Conclusions

Sequence comparison and phylogenetic analyses indicated that the South Korean PEDV isolates in this study differed genetically from previous isolates from South Korea and were most genetically similar to PEDV strains emerging in the United States during 2013. Therefore, our data suggest that the recent strains from South Korea might have originated from the United States, likely by the importation of pig breeding stock during or after the sudden emergence of PEDV in the United States. However, it remains unclear whether the US strain–like PEDVs had existed in South Korea before the emergence of PEDV in the United States. Our retrospective study, using PEDV-positive fecal samples obtained during early 2013 (KNU-1301–03), verified the presence of a PEDV isolate (KNU-1303) in South Korea in May 2013 that was placed in the same clade as the US strains (Figure 1). Thus, it is also conceivable that the strains may have already been present in South Korea as a minor lineage before the recent emergence of PEDV in the United States. Given that situation, the virus could have evolved independently by recombination, or the virus could have originated directly from China and have subsequently become dominant, leading, under suitable circumstances, to the current acute outbreak in South Korea.

Further molecular epidemiologic study is needed to find temporal and geographic evidence for the exact origin and evolution of the recent US strain–like PEDVs in South Korea. In addition, the existence of distinct PEDV lineages in South Korea suggests the potential for recombination events between different PEDV lineages or sublineages and possible cocirculation of different PEDV subgroups. During the 2010–2011 foot-and-mouth disease outbreaks, more than one third of the total pig population in South Korea was slaughtered, and since then, the importation of breeding pigs has greatly increased. However, the vaccination program for PED prevention in South Korea was not fully implemented before this importation began. Thus, it is not unexpected that the pig population appears to have a low level of immunity against PEDV and, as a result, large-scale outbreaks of PED could occur. To prevent the periodic recurrence of acute PEDV outbreaks in South Korea, a proper vaccination program should be implemented to enhance overall immunity to the virus in all stock, and strict biosecurity measures should be established. In addition, current quarantine procedures should be adequately reinforced with respect to breeding stock imported from the United States. Our findings will provide insights into a better understanding of the genetic diversity of PEDV strains and contribute to the development of more effective preventive measures against PED.

Technical Appendix Comparison of nucleotide and amino acid sequences of the spike protein genes of recent of porcine epidemic diarrhea virus isolates from South Korea and porcine epidemic diarrhea virus reference strains.
