# Supplementary material for: Outbreak-Related Porcine Epidemic Diarrhea Virus Strains Similar to US Strains, South Korea, 2013
Source: Emerg Infect Dis. 2014 Jul;20(7):1223–6. doi: 10.3201/eid2007.140294 (PMC4073847; doi:10.3201/eid2007.140294)
Supplement: Technical Appendix — Comparison of nucleotide and amino acid sequences of the spike protein genes of recent of porcine epidemic diarrhea virus isolates from South Korea and porcine epidemic diarrhea virus reference strains. [file 14-0294-Techapp-s1.pdf]

Article DOI: http://dx.doi.org/10.3201/eid2007.140294

Outbreak-Related Porcine Epidemic Diarrhea Virus Strains Similar to US Strains, South Korea, 2013

Technical Appendix

Technical Appendix Table. Pairwise comparisons of the nucleotide and amino acid sequences of the S protein genes of the re-emergent Korean PEDV isolates and PEDV reference strains

Strain	CV777	KNU-0801	MN/USA	KNU-1304	KNU-1305	KNU-1306	KNU-1307	KNU-1308	KNU-1309	KNU-1310	KNU-1311	KNU-1401	KNU-1402
CV777	-	94.0	93.7	93.4	93.7	93.5	93.8	93.8	93.7	93.4	93.5	93.4	93.5
KNU-0801	93.1	-	94.6	94.3	94.6	94.4	94.7	94.7	94.6	94.4	94.4	94.4	94.5
MN/USA	93.2	94.3	-	99.5	99.7	99.6	99.8	99.8	99.8	99.1	99.6	99.4	99.7
KNU-1304	92.5	93.5	98.9	-	99.4	99.3	99.5	99.5	99.5	98.8	99.3	99.2	99.4
KNU-1305	93.0	94.0	99.4	98.9	-	99.6	99.8	99.8	99.8	99.1	99.5	99.4	99.7
KNU-1306	92.9	93.8	99.2	98.6	99.4	-	99.7	99.7	99.6	98.9	99.4	99.3	99.5
KNU-1307	93.2	94.2	99.6	99.0	99.7	99.4	-	99.9	99.9	99.2	99.6	99.5	99.8
KNU-1308	93.2	94.3	99.7	99.1	99.7	99.4	99.9	-	99.9	99.2	99.7	99.5	99.8
KNU-1309	93.2	94.3	99.7	99.1	99.7	99.4	99.9	100	-	99.2	99.6	99.5	99.7
KNU-1310	93.0	94.2	99.2	98.6	99.1	98.8	99.2	99.3	99.3	-	98.9	98.8	99.0
KNU-1311	92.6	93.6	99.2	98.6	99.1	98.8	99.2	99.3	99.3	98.7	-	99.3	99.6
KNU-1401	92.7	93.7	98.9	98.5	99.0	98.7	99.2	99.2	99.2	98.6	98.6	-	99.4
KNU-1402	92.9	93.9	99.3	98.9	99.4	99.1	99.5	99.6	99.6	98.9	99.1	98.9	-

The percent nucleotide identity was shown in the upper right and the percent amino acid identity was presented in the lower left.
